# Comparison of sleep quality assessed by actigraphy and questionnaires to healthy subjects

**DOI:** 10.5935/1984-0063.20180027

**Published:** 2018

**Authors:** Arturo Forner-Cordero, Guilherme Silva Umemura, Fabianne Furtado, Bruno da Silva Brandão Gonçalves

**Affiliations:** 1 University of São Paulo, Biomechatronics Lab. (EPUSP) - São Paulo - SP - Brazil.; 2 Federal Institute of Education, Science and Technology of Southeast of Minas Gerais, Physical Therapy - Barbacena - MG - Brazil.; 3 Federal University of São Paulo, Psychiatry - São Paulo - SP - Brazil.

**Keywords:** Actigraphy, Surveys and Questionnaires, Sleep Deprivation

## Abstract

Sleep quality analysis is crucial for human health and it is related to duration, rhythm and quality. The goal of this study is to analyze objective assessment of the sleep-wake cycles with actigraphy, subjective questionnaires and their relationship with sleep quality indices. A wearable actigraph registered the sleep habits of 41 healthy subjects for 9 days. Afterwards, the subjects filled two questionnaires about sleep quality (Pittsburgh Sleep Quality Index) and sleepiness (Epworth Sleepiness Scale). The subjects were divided into two groups based on cut-off scores and the actigraphy parameters were compared between groups. Group 1 in ESS and PSQI categorization had less diurnal sleepiness and better sleep quality, respectively, than Group 2. Measurements of regularity (IS), fragmentation (IV), active phase amplitude (M10), rest amplitude (L5), and relative amplitude (RA) were compared between groups. Group 2 had higher L5 values. Parameter L5 (lowest of 5 consecutive hours of activity) was concluded to be relevant to identify the sleep conditions of the subjects.

## INTRODUCTION

Sleep is a complex phenomenon that could be understood and assessed using objective (polysomnography, actigraphy) and/or subjective (sleep diaries, questionnaires) approaches.

Actigraphy provides a non-invasive method to assess sleep-wake cycles over long periods, from days to months. It is based on continuously monitoring body movements and identifying the activity and resting periods. Its advantage is providing information for extended periods in the natural environment of the user^1^. The use of actigraphy is usually complemented with qualitative methods for a more complete information about sleep problems, and sleep-related behaviors^2^. For instance, questionnaires such as the Pittsburgh Sleep Quality Index (PSQI) or the Epworth Sleep Scale (ESS) have been employed as general measures of sleep quality and daytime sleepiness, and also to assess health and daytime dysfunction^3^.

In the sleep phase most of the body’s physiological recoveries occur, such as musculoskeletal recoveries, processes related to immunity, and also in memory consolidation and learning facilitation^4^^,^^5^. Thus, sleep is an active process linked to wakefulness and the study of the interaction between these two behavioral states is necessary for the understanding of all processes involved in sleep and among the main ones is the circadian oscillation.

Borbély^6^ and Daan et al.^7^ proposed a process which involves the circadian and homeostatic regulation. In this model, the sleep pressure (homeostatic process) increases exponentially from the beginning of the wake to the beginning of the sleep, recovering the sleep pressure. The homeostatic process acts together with a circadian component (circadian process) in which there are times of the day with bigger and smaller propensity to sleep. n this way, for a better understanding of sleep phenomenon, the study of sleep and circadian parameters from the actigraphy may be a good approach in order to explain multifactorial sleep disturbances.

The relation of self-reported and some objective sleep indicators (duration, latency, and efficiency) has been analyzed in the last years^8^^-^^11^. Most of these articles do not show agreement between the two measures. However, in none of them the nonparametric circadian rhythm analysis (interday stability - IS, intraday variability - IV, the least active 5-h period - L5, the most active 10-hour period - M10, relative amplitude - Ra) was used as the method for extracting circadian characteristics from the rest-activity cycle. The degeneration of the circadian timing system likely contributes to the changes in sleep. Up to date, there are no studies comparing non-parametric circadian rhythms analysis and PSQI and ESS in healthy population. The goal of this study is to analyze the objective assessment of the sleep-wake cycles with actigraphy, the standard subjective questionnaires and their relationship with sleep quality indices.

## ACTIGRAPHY

### Hardware

The actimeter used for the analysis is the ActTrust (Condor Instruments Ltda). The ActTrust device is equipped with a 3-axis accelerometer, two precision temperature sensors, one in the skin and one for the environment and a light sensor with RGB spectrum detailing. The device can monitor up to 3 months of continuous data. The devices were configured to register the activity data and to process it with Proportional Integral Mode (PIM) algorithm with a 60 seconds epoch. This algorithm filters and integrates the acceleration to obtain a measure of the user’s activity. The PIM data with 60 seconds epoch was integrated within every hour of the days generating twenty-four epochs of 3600 seconds. The resulting data was used to calculate the nonparametric parameters.

**Figure 1:**
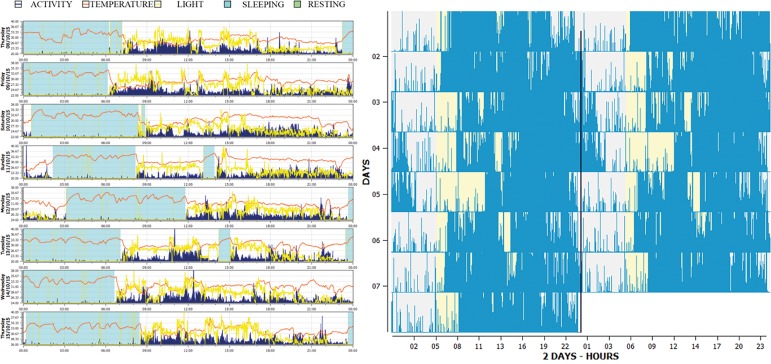
Actogram, raw actigraphy data along with the detection of the sleep intervals

The data was downloaded using the software ActStudio (Condor Instruments Ltda., SP, Brazil) (Fig. 1).

### Data analysis

The actigraphy data can be analyzed focusing sleep or the total circadian rhythm. In sleep analysis, it is possible to measure the start and end of sleep, total sleep time, latency to sleep onset, duration in minutes of nighttime awakenings, and sleep efficiency.

To assess the circadian rhythm, analyses were performed to measure regularity (IS), fragmentation (IV), amplitude of the active phase (M10), amplitude of rest (L5) and relative amplitude (RA).

The intraday variability (IV) is calculated as the average of the differences between the posterior and previous hour normalized by the variance of one-hour activity data obtained with the PIM^12^. ‘X’ is the registered activity data or registry value; ‘Xm’ is the mean of all registry values; ‘N’ is the total number of records.

(1) $$\mathrm{IV}=\frac{\sum_{i=2}^{N}{\left(X_{i}-X_{i-1}\right)}^{2}N}{\left(N-1\right)\sum_{i=1}^{N}{\left(X_{i}-X_{m}\right)}^{2}}$$

This value is used to detect fragmentation of activity rhythms. A value of high IV is usually an indicative of daytime sleep and/or nighttime awakenings.

Interday stability (IS) is calculated as the ratio of the variance of the average profile 24 hours by variance of the data. IS was calculated from the average value IS computed from 1 to 60 minutes^13^. In equation ‘p’ is the number of records in one day and ‘Xh’ is the average of all values obtained at time ‘h’ in a day.

(2) $$\mathrm{IS}=\frac{\sum_{h=1}^{p}\left(X_{h}-X_{m}\right)}{\left(p\right)\sum_{i=1}^{N}{\left(X_{h}-X_{m}\right)}^{2}}$$

As Moore-Ede et al.^14^ pointed out, changes in the IS may indicate a loose coupling between the rhythm of rest-activity and its supposedly stable markers because IS tends to decrease as the change in the day-to-day increases (that is, by definition, a weak coupling of activity patterns).

M10 is defined as the maximum sum of 10 consecutive hours of activity log. L5 is defined as the lowest sum of 5 consecutive hours of the activity log. RA is calculated as (M10-L5)/(M10+L5)^12^.

## EXPERIMENTS

### Subjects

Forty-one healthy volunteers of both genders participated in the study (mean age 22.2 years, mean body mass index 22.47 Kg/m^2^, 80.49% women). The exclusion criteria were diagnoses of psychiatric or sleep disorders.

The study was approved by the local Ethics Committee at the Federal Institute of Education, Science and Technology of the Southeast of Minas Gerais, Barbacena, Brazil (register number 39125214.0.0000.5588).

### Actigraphy

Participants were asked to wear an actimeter watch (ActTrust^®^, Condor Instruments, Brazil) all the time except while bathing. Circadian rhythm and sleep variables were collected in proportional integration mode (PIM).

### Questionnaire parameters

The Pittsburgh Sleep Quality Index Questionnaire (PSQI)^15^ subjectively evaluates sleep disturbances as well as sleep quality. This questionnaire is composed of nineteen individual items that generate seven “component” scores: subjective sleep quality, sleep latency, sleep duration, habitual sleep efficiency, sleep disturbances, use of sleeping medication, and daytime dysfunction. The score of the answers is based on a 0 to 3 scale. A global sum of the components equal or greater than “5” indicates problems in sleep.

The Epworth Sleep Scale (ESS)^16^ is a questionnaire that measures a person’s general level of daytime sleepiness. The respondents have to rate, on a 4-point scale (0 - 3), their usual chances of dozing off or falling asleep. The higher the score, the higher the person’s level of daytime sleepiness. Using a total cut-off score >10, it is possible to identify individuals with high possibility of excessive daytime sleepiness. Scores > 16 indicate severe sleepiness.

The PSQI and the ESS are two commonly employed questionnaire instruments^3^^,^^11^.

### Data analysis

The division of the Groups according to the PSQI scores was determined by the threshold of 5 points of the total scores (Group 1: total score of < 5; Group 2: score of ≥ 5). The division by ESS score was determined by the threshold of 10 points. Group 1 (n=21) in the ESS categorization has less diurnal sleepiness than Group 2 (n=20). Group 1 (n=21) in PSQI has better quality of sleep than Group2 (n=20). The normality tests were performed using the Shapiro-Wilk test and the difference of means was made using the independent T test. Statistical significance level was set at *p*≤0.05. The statistical procedures were performed using the SPSS 20 program (IBM Corp. Released 2011. IBM SPSS Statistics for Windows, Version 20.0. Armonk, NY: IBM Corp.).

## RESULTS

From the analysis of the results, significant differences are observed in L5 values by the PSQI groups categorization.

**Figure 2:**
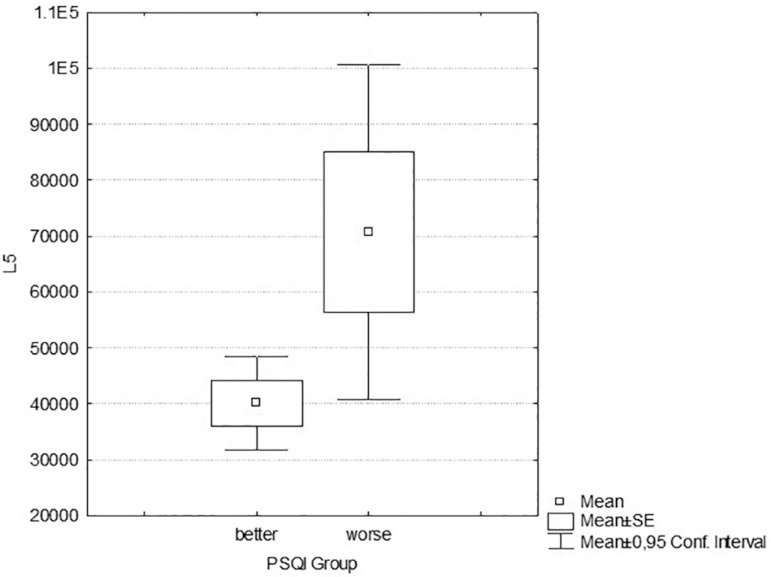
L5 values in PSQI Groups.

Individuals who have lower quality of sleep in PSQI have higher L5 values when compared with the group with better sleep quality (F=7.428; t= -2.097; *p*=0.043; Cohen’s d= -0.67; effect-size r=0.32 - Table 1, Fig. 2).

**Table 1:** Between group differences in the circadian parameters.

Circadian Parameters		Questionnaires							
		ESS- Mean (SD) and 95% CI for mean:		Upper		PSQI- Mean (SD) and 95% CI for mean:		Upper	
				Lower				Lower	
IS	Group1	.309	(0.0794)	0.273	*p* 0.23	.321	(0.0779)	0.285	*p* 0.71
				0.345				0.356	
	Group2	.343	(0.1000)	0.296		.331	(0.1040)	0.282	
				0.390				0.380	
IV	Group1	.724	(0.0939)	0.682	*p* 0.21	.731	(0.0893)	0.690	*p* 0.11
				0.767				0.771	
	Group2	.677	(0.1438)	0.609		.670	(0.1442)	0.603	
				0.744				0.738	
M10	Group1	3290422	(689010)	2976789	*p* 0.32	3183031	(615794)	2902726	*p* 0.07
				3604057				3463338	
	Group2	3570038	(1086026)	3061763		3682799	(1089727)	3172791	
				4078315				4192807	
L5	Group1	46617	(45562)	25877	*p* 0.26	40144	(18360)*	31787	*p* 0.04
				67357				48502	
	Group2	63912	(51216)	39942		70708	(64117)*	40701	
				87883				100717	
Ra	Group1	.972	(0.0226)	0.962	*p* 0.22	.974	(0.0132)	0.968	*p* 0.11
				0.982				0.980	
	Group2	.963	(0.0263)	0.950		.961	(0.0318)	0.946	
				0.975				0.976	

* p<0.05 in independent T test.

However, there are no differences when comparing the groups separated by the ESS categorization. No other statistical differences were found.

## DISCUSSION AND CONCLUSIONS

The chronic restriction is related with the contemporary daily habits; the delay in sleep due artificial lighting in the residences and waking up early due to social obligations, result in a significant reduction in quality of life^17^. Chronic sleep deprivation is associated with several health and brain function problems, which cause loss of performance in daily tasks and in the learning process^4^.

It seems that a large portion of the population is sleep deprived. The pattern of restriction and extension of sleep duration due the differences in routines on workdays and on free days generates sleep deprivation^18^^-^^20^. This pattern of restriction and extension of sleep was popularized when Wittman et al.^18^ and Roenneberg et al.^20^ adopted the term “social jet lag” to describe the same phenomenon. The authors associated social jet lag with the incidence of various diseases, such as depression, substance dependence, such as smoking and disorders, such as obesity. In addition, it cognitive deficit has been reported due to a disturbance in frontal cortex connectivity caused by sleep deprivation. Despite all of the problems caused by sleep deprivation it is difficult to measure in long-term recordings. This has led to evaluation by means of questionnaires that suffer from the subjective perception about sleep and sleep condition. The use of actigraphy has opened a window to assess activity, related to sleep, in long-term recordings. When comparing the actigraphy parameters with the ESS and PSQI questionnaires scales we have used a classification of the participants in two groups based on the questionnaires scores.

Afterwards, we have compared the actigraphy parameters from each group in order to see if they corresponded to different populations. We found that the L5 parameter obtained with the PIM algorithm was the only one that had a significant difference between the two groups defined by PSQI. The PSQI is a subjective approach to assess disturbances related to sleep and quantify sleep quality. In this way, if it is possible to measure the sleep movements in the sleep phase, it may be possible to identify an objective correlate for the sleep quality measures obtained by questionnaires. For this goal, the analysis of the actigraphy parameters is a promising approach.

Regarding the sleep deprivation assessment by sleep/wake parameters, higher values of L5 means that the subject has more awakenings or movements during the sleep phase, which may compromise the quality of rest, resulting in more diseases and disturbances related to sleep deprivation. Higher values of L5 are also observed in patients with neurological diseases, such as Alzheimer and Parkinson^13^, populations in which the quality of sleep is known to be deteriorated. However, in our analysis, differences in parameters of stability and fragmentation of sleep (IS and IV, respectively) were not observed, which leads to suppose that the phenomenon is too complex to be explained with a single parameter.

The results from the questionnaires, a subjective evaluation, match the results from the actigraphy only in one sleep/wake rhythm parameter, which is considered an objective assessment of the sleep condition of the subject. Since L5 records the movement of the individuals during the sleep phase, the use of this parameter as a criterion for the identification of sleep disturbances can contribute to a better understanding of this phenomenon.

The actigraphy data contained measurements obtained during a period of time, comprising weeks or up to months (nine days in our experiments). On the other hand, the questionnaires are usually skewed towards more recent information, probably reflecting the behavior of the last couple of days or even considering recent unexpected events that are mostly remembered.

It seems that sleep evaluation must be treated as a multivariate time series because several variables must be considered to describe sleep deprivation conditions in certain periods of time. In addition, several questionnaires capturing different aspects of sleep behavior should be taken into account. Therefore, L5 that is an actigraphy parameter and a sleep rhythm measurement, also contains information about the quality of sleep, as shown by its relationship with the sleep quality scores obtained from the questionnaires.

## References

[1] Martin JL, Hakim AD (2011). Wrist Actigraphy. Chest.

[2] Sadeh A (2015). Sleep assessment methods. Monogr Soc Res Child Dev.

[3] Mondal P, Gjevre JA, Taylor-Gjevre RM, Lim HJ (2013). Relationship between the Pittsburg Sleep Quality Index and the
Epworth Sleepiness Scale in a sleep laboratory referral
population. Nat Sci Sleep.

[4] Alvarez GG, Ayas NT (2004). The impact of daily sleep duration on health: a review of the
literature. Prog Cardiovasc Nurs.

[5] Killgore WD (2010). Effects of sleep deprivation on cognition. Prog Brain Res.

[6] Borbély AA (1982). A two process model of sleep regulation. Hum Neurobiol.

[7] Daan S, Beersma DG, Borbély AA (1984). Timing of human sleep: recovery process gated by a circadian
pacemaker. Am J Physiol.

[8] Segura-Jiménez V, Camiletti-Moirón D, Munquía-Izquierdo D, Álvarez-Gallardo IC, Ruiz JR, Ortega FB (2015). Agreement between self-reported sleep patterns and actigraphy in
fibromyalgia and health women. Clin Exp Rheumatol.

[9] Jackowska M, Ronaldson A, Brown J, Steptoe A (2016). Biological and psychological correlates of self-reported and
objective sleep measures. J Psychosom Res.

[10] Lemola S, Ledermann T, Friedman EM (2013). Variability of sleep duration is related to subjective sleep
quality and subjective well-being: an actigraphy study. PLoS One.

[11] Buysse DJ, Hall ML, Strollo PJ, Kamarck TW, Owens J, Lee L (2008). Relationships Between the Pittsburgh Sleep Quality Index (PSQI),
Epworth Sleepiness Scale (ESS), and Clinical/Polisomnographic Measures in a
Community Sample. J Clin Sleep Med.

[12] Witting W, Kwa IH, Eikelenboom P, Mirmiran M, Swaab DF (1990). Alterations in the circadian rest-activity rhythm in aging and
Alzheimer’s disease. Biol Psychiatry.

[13] Gonçalves BS, Cavalcanti PR, Tavares GR, Campos TF, Araujo JF (2014). Nonparametric methods in actigraphy: An update. Sleep Sci.

[14] Moore-Ede MC, Sulzman FM, Fuller CA (1982). The Clocks That Time Us: Physiology of the Circadian Timing
System.

[15] Buysse DJ, Reynolds CF, Monk TH, Berman SR, Kupfer DJ (1989). The Pittsburgh Sleep Quality Index: a new instrument for
psychiatric practice and research. Psychiatric Res.

[16] Johns MW (1991). A new method for measuring daytime sleepiness: the Epworth
sleepiness scale. Sleep.

[17] Roenneberg T, Wirz-Justice A, Merrow M (2003). Life between clocks: daily temporal patterns of human
chronotype. J Biol Rhythms.

[18] Wittmann M, Dinich J, Merrow M, Roenneberg T (2006). Social jetlag: misalignment of biological and social
time. Chronobiol Int.

[19] Korczak AL, Martynhak BJ, Pedrazzoli M, Brito AF, Louzada FM (2008). Influence of chronotype and social zeitgebers on sleep/wake
patterns. Braz J Med Biol Res.

[20] Roenneberg T, Allebrandt KV, Merrow M, Vetter C (2012). Social jetlag and obesity. Curr Biol.

